# Genetic Diversity and Population Structure of Nutria (*Myocastor coypus*) in South Korea

**DOI:** 10.3390/ani9121164

**Published:** 2019-12-17

**Authors:** Il Ryong Kim, Wonkyun Choi, Areum Kim, Jongpyo Lim, Do-Hun Lee, Jung Ro Lee

**Affiliations:** 1 National Institute of Ecology (NIE), Seocheon 33657, Korea; kir6060@nie.re.kr (I.R.K.); wonkyun@nie.re.kr (W.C.); arkim@nie.re.kr (A.K.); 2 Division of Applied Life Science and PMBBRC, Gyeongsang National University, Jinju 52828, Korea; 3 Department of Environmental and Occupational Health Sciences, University of Washington, Box 357234, Seattle, WA 98195, USA; imjoe507@gmail.com

**Keywords:** nutria, *Myocastor coypus*, invasive species, genetic diversity, population structure

## Abstract

**Simple Summary:**

Nutrias (*Myocastor coypus*) are large semiaquatic rodents native to subtropical and temperate South America. Nutrias have been introduced on all continents, except Oceania and Antarctica, and have become invasive in many countries. This study carried out a survey of nutrias in South Korea under the Nutria Eradication Project from January 2013 to August 2015. Out of 1509 habitat traces, 1497 tracks were observed in the Nakdong River basin. Based on the ecological field data, we identified concentrated areas of nutria populations. Tissue samples were collected from captured nutria for genetic analysis. According to the microsatellite marker analysis, the estimated genetic diversity of the nutria populations was low, which suggests that nutrias in South Korea originate from a single population.

**Abstract:**

The nutria (*Myocastor coypus*) is an invasive alien species that have had major adverse effects on biodiversity and the agricultural economy in wetland habitats. Since 2014, the Ministry of Environment in South Korea has been carrying out the Nutria Eradication Project, and we investigated nutria distribution and genetic diversity of nutria populations in South Korea. We estimated that 99.2% of nutria habitats are in the mid-lower Nakdong River regions. To further analyze the genetic diversity in eight major nutria populations, we performed a genetic analysis using microsatellite markers. Genetic diversity levels of the eight nutria populations in South Korea were relatively lower than those in other countries. The probability of migration direction among nutria populations was predicted from genetic distance analysis. Genetic structure analysis showed little difference among the nutria populations in South Korea. These results suggest that nutrias in South Korea originated from a single population. Our results provide important data for establishing management strategies for the successful eradication of nutria populations in South Korea, as well as in other countries with alien invasive species.

## 1. Introduction

Environmental concerns regarding invasive alien species have been rapidly increasing with the expansion of global trade. Invasive mammalian species, especially rodents, have adverse effects on the biodiversity of wetlands, such as reducing biodiversity, disrupting ecosystems, human activities, and human welfare [1,2]. The nutria (*Myocastor coypus*) is a large, semi-aquatic rodent that originates from the southern regions of South America and has been introduced to every continent except Oceania and Antarctica [3,4,5,6]. In addition, nutria populations have increased rapidly in ecosystems where the nutria was introduced and caused damage to the natural environment, crop production, and irrigation systems [7,8,9,10,11,12,13]. Previously, to elucidate information regarding nutria, many studies have investigated habitat selection, behavior, migration, breeding, feeding, and interaction patterns with other species in both native and introduced nutria populations [14,15,16,17,18].

Nutrias were first introduced to South Korea in 1985. Commercial nutria farming was initiated in 1987, and the number of nutria breeding farms rapidly increased to 450 by the early 2000s [6,19]; the nutria farming business then began declining in scale. Since then, nutria individuals have been unintentionally released from unmanaged farms into the natural environment. These escaped individuals formed a settlement of wild nutria populations in South Korea. The Korean Ministry of Environment began investigating the status of nutrias in 2006 and designated the nutria as an invasive alien species in 2009. Previous studies reported that the settlement of nutrias in South Korea is concentrated in the Nakdong River basin [19,20,21,22].

To reduce the number of nutrias in the natural environment, the Korean government implemented The Nutria Eradication Project in 2014. Several government bodies are currently involved in this program—including the Ministry of Environment, local environmental agencies, the National Institute of Ecology (NIE), local governments, and private organizations. Furthermore, the Ministry of Environment organized a strategic committee to establish relevant policies and detailed plans [22]. The local offices hired hunters to capture nutrias throughout the year and the NIE supported eradication strategies and techniques. Due to these efforts, 27,487 nutrias were captured in the Nakdong River basin from 2016 to 2018 [22].

Prior to the initiation of the Nutria Eradication Program, the Korean government focused on the three strategic approaches: (1) monitoring of nutria individuals and habitats; (2) predicting the population distribution by analyzing the genetic structure; and (3) developing eradication programs based on nutria ecology. In a recent study, Kim and colleagues have focused on characterizing habitat location and distribution patterns to provide fundamental data for estimating habitat change in South Korea [22]. However, not enough is known about the genetic information of nutrias to estimate the relationship among nutria populations in South Korea.

To improve the nutria eradication program, genetic information on populations in highly concentrated habitats near the Nakdong River basin is needed to estimate migration patterns for preventing the re-colonization of nutria. Microsatellite markers are broadly available and have been applied to study the population structures and dynamics in many invasive rodents, such as nutrias, ship rats, and Norway rats [23,24,25,26,27]. Eradication programs have failed due to the lack of ecological and population genetic information on target invasive species [28,29,30,31]. Information about population genetics allows estimating and predicting the relationship between the target populations and neighboring populations of invasive species. Analyzing the genetic diversity and population structure can help predict dispersal and ranges of populations [28,32,33,34]. In this study, we carried out a survey of nutrias in South Korea and collected tissue samples for genetic analysis from captured nutrias in eight different regions. Our results can be applied to successful invasive nutria management for long-term eradication projects in South Korea.

## 2. Materials and Methods

### 2.1. Survey and Tissue Sampling

**Figure 1:**
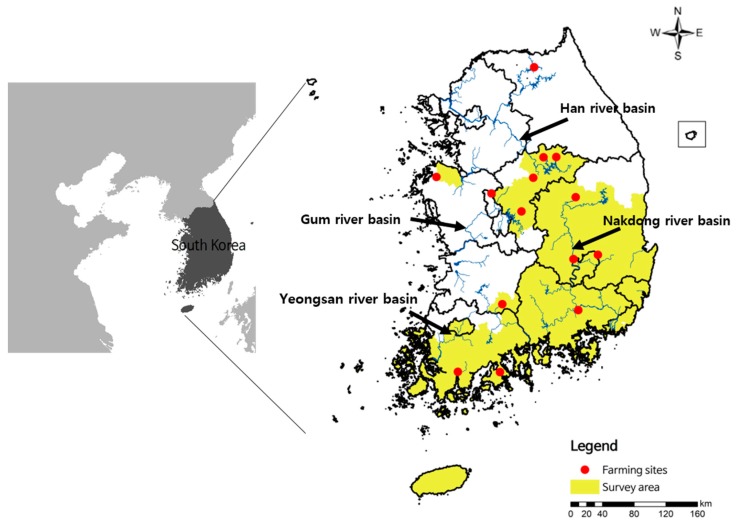
Maps of the nutria survey areas. Red dots represent nutria farming sites (2002–2013) and the yellow-colored area is the survey area in this study.

The nutria distribution investigation was conducted in South Korea from January 2013 to August 2015 based on the information on nutria breeding farm locations and four watershed basins: the Han River, Geum River, Yeongsan River, and Nakdong River basins. The locations of the farms, survey area, and tissue sample collection sites are presented in Figure 1. Nutria feces, footprints, bank holes, and nests, as well as active nutria individuals, were observed by surveyors in the watersheds around the catchment area. Confirmed sites of nutria habitation coordinates were recorded using GPS (GPS V; Garmin Inc., Olathe, KS, USA). To analyze the distribution of concentrated nutria habitats, we used ArcGIS 10.3.1 (http://pro.arcgis.com) and observed habitat traces within a 100 m × 100 m area (1 ha) which were visualized by one habitat trace [19,21]. This means that the observed habitat traces per 1 ha were indicated as one dot in the nutria distribution map.

Nutria habitats were also analyzed using the kernel density tool, which utilizes kernel functions to estimate the degree of concentration per unit area, in ArcGIS 10.3.1. The kernel density tool was used based on the method described by Silverman [35]. The kernel density estimation undergoes a smoothing phase of location coordinate data. Defining the bandwidth of a one-sided kernel function affects the probability density function estimation [36,37]. Methods to adjust the bandwidth include LSCVh (least squares cross-validation), CVh (cross-validation), and h_ref (reference method). As adequate bandwidth values may differ depending on the distribution of points [38], the default setting in the search radius in the kernel density tool was used (5485.4 m). The calculated LSCVh, Cvh, and h-ref values were applied accordingly using Animal Space Use 1.3 Beta. Kernel density was calculated using bandwidth calculations based on nutria habitat traces (Cvh 1018.4 m; LSCVh 5.8 m; h_ref 7293.5 m; default search radius value 5424.2).

**Figure 2:**
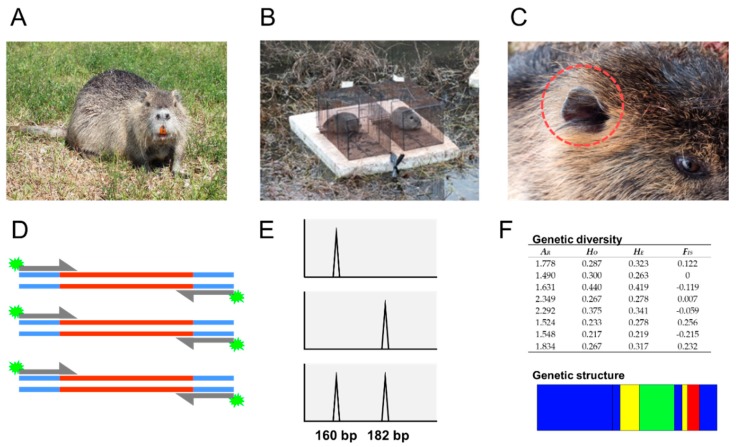
Procedure for DNA extraction and microsatellite genotyping from nutria individuals. (**A**) nutria in nature, (**B**) cage traps of nutria, (**C**) nutria tissue sampling for DNA extraction (red circle), (**D**) amplification of microsatellite region using fluorescent-labeled primer sets, (**E**) analysis of amplified fragment, (**F**) examples of results of genetic diversity and genetic structure.

Nutria tissue samples (e.g., ears or tails) were collected from eight regions containing large nutria populations in the Nakdong River basin (Figure 2A–C): Busan metropolitan city (BUS, 39 individuals), Changwon city (CHA, 4 individuals), Daegu metropolitan city (DAE, 10 individuals), Jinju city (JIN, 18 individuals), Gimhae city (GIM, 4 individuals), Oesan-ri, Miryang city (MIO, 3 individuals), Yuldong-ri, Miryang city (MIY, 6 individuals), and Yangsan city (YAN, 9 individuals) (Table 1).

### 2.2. DNA Extraction and Microsatellite Genotyping

Genomic DNA was extracted using a QIAamp DNA mini kit (QIAGEN, Germany) according to the manufacturer’s instructions and DNA concentrations were measured by NanoDrop (Thermo Scientific, Wilmington, DE, USA). To analyze the microsatellite variability, 100 ng/µL DNA was used and 12 microsatellite markers (*McoD214*, *McoD271*, *McoD59*, *McoD69*, *McoC124*, *McoC203*, *McoD60*, *McoD60*, *McoB17*, *McoC118*, *McoA04*, and *McoD228*) were amplified with fluorescent-labeled primer sets (Figure 2D–F) [26,39]. Amplified fragments were analyzed by an ABI3130XL Genetic Analyzer (Applied Biosystems, USA) and the data were scored using GeneMarker v2.6.4 (SoftGenetics LLC, State College, PA, USA) (Figure 2G).

### 2.3. Genetic Data Analysis

Expected heterozygosity (*H_E_*) and observed heterozygosity (*H_O_*) were estimated using GenAlEx v6.5 [40]. FSTAT v2.9.3 (http://www2.unil.ch/popgen/softwares/fstat.htm) was also used to calculate the inbreeding coefficient (*F_IS_*) and allelic richness (*A_R_*) among populations and loci [41]. Analysis of Molecular Variance (AMOVA), which incorporates molecular differences at two hierarchical levels (within and among populations), for population differentiation among nutria populations in the Nakdong River basin was performed using Arlequin 3.5 (http://cmpg.unibe.ch/software/arlequin35/) [42,43]. To investigate population structure, a Bayesian model-based clustering method was applied using STRUCTURE version 2.3.4 (https://web.stanford.edu/group/pritchardlab/structure.html) with *K* ranging from 1 to 10 and 100,000 Markov chain Monte Carlo (MCMC) steps under an admixture model. The value of Δ*K* was calculated using the method described by Evanno [44]. The population structure was investigated in finer detail using Bayesian Analysis of Population Structure (BAPS) 5.4 (http://www.helsinki.fi/bsg/software/BAPS/) [45,46,47]. The pairwise population *F_ST_* values of genetic divergence and Nei’s genetic distance were calculated using the statistical program GenAlEx v6.5 (http://biology-assets.anu.edu.au/GenAlEx/Welcome.html) [40,48]. A Mantel test determined by the TFPGA 1.3 program (https://www.mybiosoftware.com/tfpga-1-3-analysis-allozyme-molecular-population-genetic-data.html) was performed to identify correlations between geographical distance and genetic distance among the nutria populations [49].

## 3. Results and Discussion

### 3.1. Nutria Distribution and Tissue Sampling

**Figure 3:**
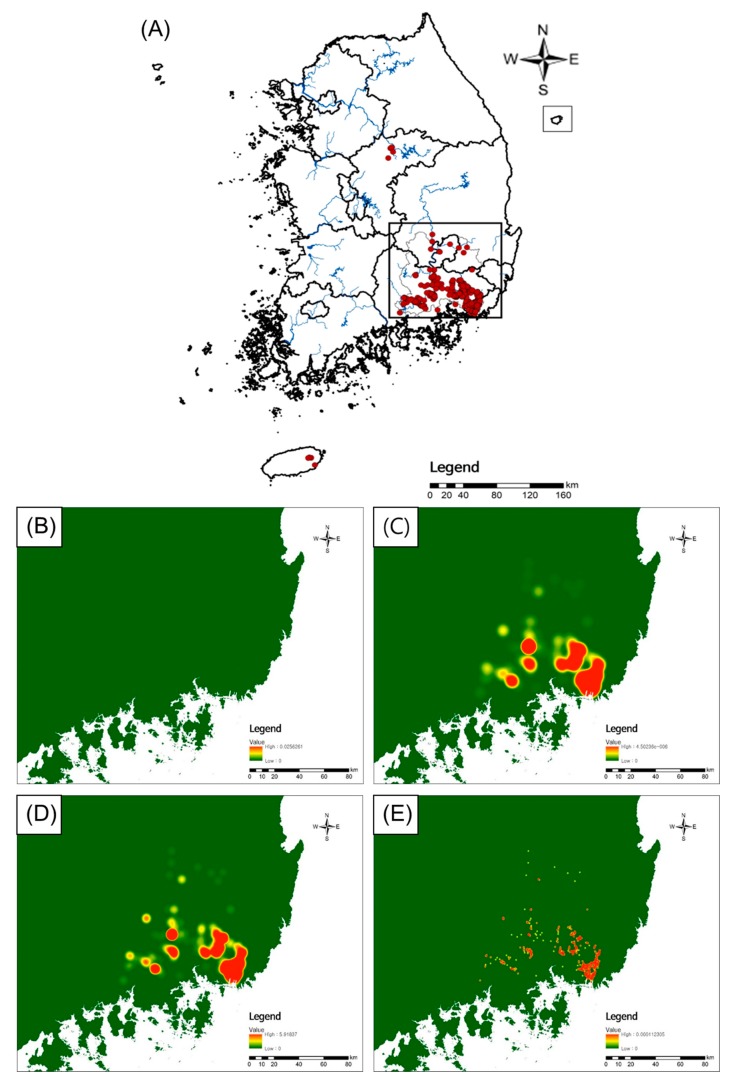
Kernel density using four different radii. (**A**) Red dot: habitat traces; (**B**) default: using the default value for kernel function bandwidth; (**C**) h_ref: calculated by the reference method used for kernel function bandwidth; (**D**) LSCVh: calculated by Least Squares Cross-Validation used for kernel function bandwidth; (**E**) CVh: calculated by Cross-Validation used for kernel function value.

Representative habitat traces of nutria distribution were observed in two water systems in the Han River and Nakdong River basins and one in Jeju Island. Among the 1509 habitat traces, 1497 were observed in the Nakdong River basin, five in the Han River basin, and seven in Jeju Island (Figure 3A).

**Figure 4:**
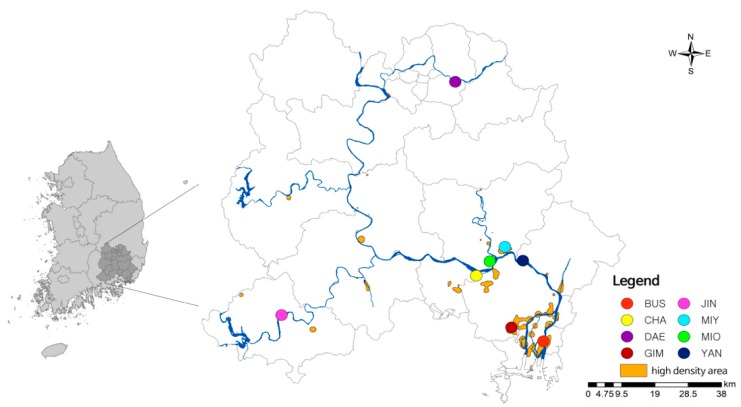
Locations of sample collection sites from high-density nutria habitat tracks. CVh 0.1% high-density area (orange-colored region).

The kernel density was used to estimate a smoothing phase of location coordinate data. Nutria samples in the 0.1% high density distributed area were collected using the CVh value in the kernel density calculation (Figure 3E). The ecological and scenery conservation areas were disregarded as viable sample collection sites, as wildlife capture in the areas is restricted by the National Environment Conservation Act of South Korea. Sample collection sites in Daegu and Jinju at the northern and western ends were included based on habitat track evidence. The majority of observed habitat traces in this study were observed in the middle and lower regions of the Nakdong River basin (99.2%). These results show that the Nakdong River basin is the most populated area for nutria individuals in South Korea. In line with previous nutria distribution studies in South Korea, the kernel density results support the middle and lower reaches of the Nakdong River having slow water flow leading to tributaries and wetlands, with a warm winter and abundant food sources, providing a good habitat for nutria populations to establish and proliferate [19,50]. A previous investigation reported that nutria management efforts are focused on the Nakdong River basin in South Korea because nutria individuals are mainly clustered in this region [22]. Although the previous management was effective to decrease nutria habitat tracks, we tried to apply genetic analysis for the developed nutria eradication project in South Korea. According to nutria distribution analysis (Figure 3 and Figure 4), we selected eight regions (BUS, CHA, DAE, JIN, GIM, MIO, MIY, and YAN) of nutria habitat containing large nutria populations in the middle and lower reaches of the Nakdong River basin for genetic analysis (Table 1, Figure 4). A total of 93 nutria individuals were captured and genomic DNA used for genetic density analysis.

### 3.2. Genetic Diversity Analysis of Nutria Populations

Previous studies have reported successfully settled nutria populations in the middle to lower reaches of the Nakdong River basin, as well as in other sites, such as the Han River and Jeju Island [19,50]. Estimations of the patterns of migration among nutria populations may help the eradication programs prevent the re-colonization of nutria [26]. However, information on the genetic diversity among populations is unknown for nutrias in South Korea. As a result of the analysis of microsatellite markers, all microsatellite markers in the nutria populations were polymorphic except for *McoD228*, which had no heterozygosity. Some microsatellite markers were presented as monomorphic within populations, namely *McoD59* and *McoC203* in CHA, *McoC203* in JIN, *McoD214* in MIO, and *McoC203* and *McoD60* in MIY. These results indicate that the genetic diversity among nutria populations in South Korea is relatively low.

Invasive species, established in a relatively small population, can undergo bottlenecks that may decrease its genetic diversity [51]. Genetic diversity monitoring of invasive species can provide information about invasion history and possible management strategies [52]. Allelic richness (*A_R_*) and observed heterozygosity (*H_O_*) are generally used to estimate the genetic diversity among populations [23,26,27,34,39,53]. To evaluate the genetic diversity among nutria populations, we calculated allelic richness (*A_R_*), observed heterozygosity (*H_O_*), and expected heterozygosity (*H_E_*). The average values of *A_R_, H_O_*, and *H_E_* were 1.806, 0.298, and 0.305, respectively (Table 2). The values of genetic diversity were affected by the size of the nutria population; however, the genetic diversity of South Korean nutrias did not correlate with the nutria population size (R^2^ = 0.0137). Nutria populations in South Korea had relatively lower levels of *H_O_* (0.298) than those from other locations, such as Louisiana, USA (0.624, 9 markers) [27]; Czech Republic (0.504, 11 markers) [39]; Maryland, USA (0.46, 27 markers) [26]; and the Argentinean Pampas (0.658, 16 markers) [53]. Furthermore, nutrias in South Korea had lower *A_R_* values (1.806) than those in other locations, such as Louisiana, USA (3.8) [27] and the Czech Republic (3.6) [39]. These low levels of *H_O_* and *A_R_* indicate that nutria in the Nakdong River basin have a low level of genetic diversity. The low level of genetic diversity and lack of heterozygosity in the *McoD228* marker may suggest that nutria in South Korea spread from one population source. This means that, even though nutrias were imported twice, only the secondary import from Bulgaria successfully settled in the Nakdong River, and the population from France [6] did not.

As the populations are quite small and isolated, inbreeding within populations can be calculated to be a factor in the low genetic diversity that we observed [54]. The inbreeding coefficients (*F_IS_*) were calculated to estimate the population size and possibility of migration. The calculated *F_IS_* values were 0.256 and 0.232 in the MIO and YAN populations, respectively (Table 2). The MIO and YAN populations were distributed near small reservoirs and isolated from other populations by a road and villages. Negative *F_IS_* values were observed in the DAE, GIM, and MIY populations, indicating that there is outbreeding in these populations. The net-like stream as a topographical factor in these areas can allow easy movement of the nutria. Although BUS has the best settlement conditions, the area is geologically separated from other habitats by many highway roads and a well-developed estuary, and therefore the nutrias underwent many cases of inbreeding in small habitats. These values support the theory that nutria populations originated from a small number of individuals, and that the populations are isolated (Table 2).

### 3.3. Population Structure Analysis of Nutria Populations

Despite the technical advances in the invasive mammal eradication program, molecular genetics knowledge has not been fully integrated into these control programs. Although the source of Korean nutrias was estimated, *F_IS_* values suggest that the environmental conditions of each population were different. Different colonizing populations had a high genetic structure. Therefore, those populations had capacities to promote invasiveness [55]. AMOVA, STRUCTURE, and BAPS are generally used to estimate the genetic structure of a population. To elucidate the difference in each nutria population, the population structure was analyzed using AMOVA, STRUCTURE, and BAPS. AMOVA, which is a statistical analysis for molecular differences with two hierarchical levels, indicated that among-population variation only accounted for 10.7% of the total genetic variation, whereas within-population variation explained 89.3% (Table 3). These results suggest that there is no significant differentiation among the nutria populations in South Korea.

**Figure 5:**
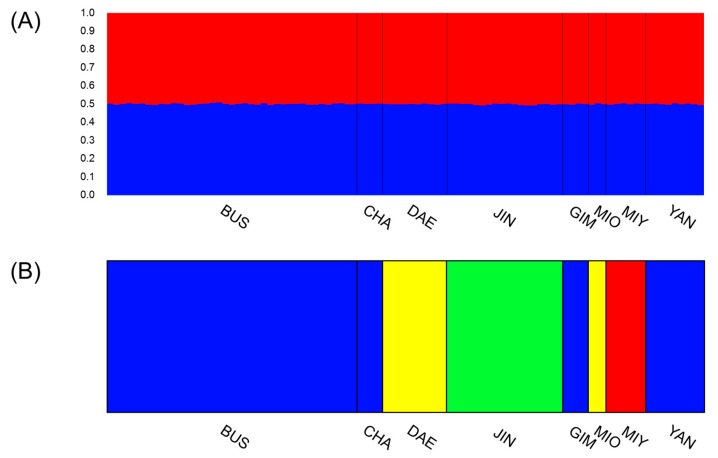
Nutria population structure analysis results. Each population is separated by a vertical bar. Abbreviations for populations are shown under panels. (**A**) STRUCTURE bar plot for K = 2, showing the same structures in eight populations; (**B**) Four groups identified by BAPS. Group 1: BUS, CHA, GIM, and YAN; Group 2: BAE and MIO; Group 3: JIN; Group 4: MIY.

To confirm these results, STRUCTURE using Bayesian clustering was conducted. Each bar represents an individual nutria and each population is separated by vertical bars. K = 2 and the optimal number of clusters was calculated using Evanno’s method. Populations show similar assignment probabilities. STRUCTURE results showed a similar trend to the AMOVA pattern that each nutria population had a similar structure (K = 2) (Figure 5A). BAPS, using a more sensitive clustering algorithm, was used to analyze the population structure among nutria populations in detail. BAPS identified four groups: (1) BUS, CHA, GIM, YAN; (2) DAE, MIO; (3) MIY; and (4) JIN. Each population separated by a vertical bar represents a different color depending on the group. These groups are not correlated with their geographic distribution (Figure 5B). These patterns assumed that there was an artificial change in the gene pool from nutria farms near DAE and JIN in the past. According to the results of BAPS, there was a little difference among nutria populations; however, AMOVA and STRUCTURE data showed that populations of nutria in South Korea had similar structures and also indicated that nutria populations in Korea originate from one source.

### 3.4. Genetic Distance of Nutria Populations

Pairwise genetic distances (*F_ST_*) show the degree of genetic relationship between populations. *F_ST_* has been used to infer migration rates and represent genetic differentiation among populations [56]. To confirm the relationship between the eight nutria populations, we calculated the *F_ST_* of nutrias in South Korea. A low value of *F_ST_* between two populations means similar genetic structures and little population differentiation. We estimated the values of *F_ST_* using eight nutria populations in South Korea. The values varied from 0.013 between the BUS and YAN populations to 0.292 between the JIN and MIO populations (Table 4). Some populations, such as CHA/MIY and MIO/MIY, are separated by a short geographical distance but are more distantly related from a genetic point of view than other pairs of populations. Although a Mantel test was applied to elucidate the relationship between genetic distance and geographical distances, the data showed no significance (r = 0.28). The current distribution of nutria populations may have been influenced by humans.

The analysis of *F_ST_* identified the same four groups of populations as BAPS. Nutria individuals that belong to BUS, CHA, GIM, and YAN populations may migrate and interbreed along the Nakdong River. However, the high value of *F_IS_* from YAN suggests that the populations likely inbreed with closely related individuals and the population sizes are extremely small (Table 2). The YAN population may be descended from the BUS population, as they are both close to the Nakdong River. Low values of *F_ST_* (0.022) between DAE and MIO imply that nutrias can migrate between the two regions through the Miryang River and the Cheongdo Stream. As shown in Table 2, low values of *F_IS_* (-0.119) in the DAE population imply that the population may have established a sufficiently large population for outbreeding, whereas the MIO population may have a relatively small population according to the high value of *F_IS_* (0.256), as shown in Table 2. The JIN and MIY populations had the greatest genetic distances of all the populations. The MIY population had a high level of FST compared to other populations, including the MIO population, even though these two populations are geographically close. The genetic divide in the MIY population may have been facilitated by the isolated habitat near the reservoir. However, based on the negative value of *F_IS_*, the MIY population may have established a sufficiently large population for outbreeding (Table 2).

From our results, we confirm nutria population groups in the Nakdong River basin and decided that capturing each nutria group using genetic data on migration direction in the area would be a more efficient approach than a broader eradication strategy that does not use prediction data. Having implemented this strategy to effectively capture individual nutria groups, the Government started operating additional maneuver capture teams, as well as the existing nutria capture teams in the assigned Nakdong River basin area. These strategic modifications from the primary plans have enabled a more efficient nutria elimination strategy in South Korea that reduced 88% of the nutria habitat from 2014 to 2018 [22].

## 4. Conclusions

We estimated the genetic origin of and relationships among nutria populations that successfully settled in South Korea as an invasive alien species. The Nakdong River hydrosphere is densely interconnected and resembles a net-like structure. This prevents the complete eradication of nutria, despite the progress in nutria capture techniques and ongoing efforts at the national level. For the successful nutria eradication project, fundamental genetic information on nutria population dispersion and habitat changes with respect to the Nakdong River hydrosphere is necessary for South Korea. In this study, genetic diversity and structural analysis indicated that the populations of nutria in the middle-low regions of the Nakdong River might originate from one population source, possibly from the second nutria import, and the populations can be classified into four groups using pairwise genetic distance data. These genetic analyses can be used to estimate the population structure and genetic diversity of invasive species in general, especially vertebrates. We investigated the establishment process and the population composition of nutria prior to the initiation of the Nutria Eradication Project. Using genetic diversity analysis, population structure and geographical migration were inferred, establishing a foundation for estimating habitat change and creating detailed long-term eradication plans.

## Figures and Tables

**Table 1:** Information on nutria samples in the Nakdong River basin of South Korea.

Population ID	*N*	Coordinate	Sampling Site (Address)
BUS	39	35°09′15.7″ N 128°57′21.9″ E	Busan metropolitan city
JIN	18	35°14′7.32″ N 128°8′16.68″ E	Jinju city
DAE	10	35°51′46.5″ N 128°42′43.7″ E	Daegu metropolitan city
YAN	9	35°22′21.3″ N 128°53′49.5″ E	Yangsan city
MIY	6	35°24′38.1″ N 128°50′29.2″ E	Miryang city (1)
CHA	4	35°20′2.66″ N 128°44′59.76″ E	Changwon city
GIM	4	35°11′35.6″ N 128°51′26.2″ E	Gimhae city
MIO	3	35°22′20.88″ N 128°47′34.95″ E	Miryang city (2)

*N*: number of nutria individuals.

**Table 2:** Estimated nutria sample sizes and genetic diversity of eight populations in South Korea.

Population ID	*N*	*A_R_*	*H_O_*	*H_E_*	*F_IS_*
BUS	39	1.778	0.287	0.323	0.122
CHA	4	1.490	0.300	0.263	0
DAE	10	1.631	0.440	0.419	−0.119
JIN	18	2.349	0.267	0.278	0.007
GIM	4	2.292	0.375	0.341	−0.059
MIO	3	1.524	0.233	0.278	0.256
MIY	6	1.548	0.217	0.219	−0.215
YAN	9	1.834	0.267	0.317	0.232

*N*: number of individuals. *A_R_*: allelic richness. *H_O_*: observed heterozygosity. *H_E_*: expected heterozygosity. *F_IS_*: inbreeding coefficients.

**Table 3:** Summary of AMOVA within/among eight nutria populations.

Analysis	Source of Variation	d.f.	Variance Component	% of Variation	*p*
Nutria population	Among populations	7	0.190	10.7	<0.001
	Within populations	178	1.583	89.3	<0.001
	Total	185	1.773	100	

d.f.: degrees of freedom.

**Table 4:** Genetic distances among eight nutria populations using pairwise genetic distance (*F_ST_*).

Populations	BUS	CHA	DAE	JIN	GIM	MIO	MIY	YAN
BUS	-	0.017	0.073	0.147	−0.044	0.062	0.158	0.013
CHA	-	-	0.082	0.109	0.017	0.061	0.268	−0.041
DAE	-	-	-	0.18	0.03	0.022	0.122	0.096
JIN	-	-	-	-	0.194	0.221	0.292	0.155
GIM	-	-	-	-	-	0.048	0.094	−0.029
MIO	-	-	-	-	-	-	0.239	0.074
MIY	-	-	-	-	-	-	-	0.179
YAN	-	-	-	-	-	-	-	-
